# Carleton’s Genito-Urinary and Veneral Diseases

**Published:** 1895-12

**Authors:** 

**Affiliations:** 497 Fifth Ave., New York


### CARLETON'S GENITO-URINARY AND VENEREAL DISEASES.

497 Fifth Ave., New York, October 15th, 1895.

The Homœopathic Physician, Philadelphia, Pa.:

We are to-day in receipt of The Homœopathic Physician for September, with the review of *Carleton's Genito-Urinary and Venereal Diseases* therein. We think that the review was very superficial, and what is printed is very misleading to the profession. We desire to set you right in the matter, and hope you will give your readers the benefit of the same. It states, "in speaking of the treatment of gonorrhœa in the male, lists of remedies are given, but no indications of them." We desire to call your attention to page 64 of said book, and following pages up to page 70, inclusive. These pages contain nothing but the indications of homœopathic remedies in gonorrhœa. Said review states, "referring to chancroids, the indications for homœopathic remedies are absent." For this subject, we beg to call your attention to the bottom of page 211, where reference is made for special indications in chancroids to pages 26 to 33, which contains nothing but indications for homœopathic remedies. Again, concerning syphilis, we desire to call your attention to pages 295 to 300, which contain nothing but the indications of homœopathic remedies for syphilis.

Believing that it was not your intent to mislead the physicians as to the contents of the book or its merits, we have, at the suggestion of the author, called your attention to these matters mentioned above, and trust you will make some little mention in your journal giving these facts. It is the plan of a book, after every heading of disease or ailment to follow the same with a chapter, giving a special therapy of homœopathic remedies, which it is evident that your critic has overlooked.

We remain, Yours very truly,

Boericke, Runyon & Ernesty.

[The above statement concerning Dr. Carleton's book is perfectly accurate, and The Homœopathic Physician stands corrected. We publish the correction with pleasure, as we have no idea of being unjust to any one.—Ed.]

